# Mental Health Consequences of the July Revolution in Bangladesh: A Study on Depression and Post-traumatic Stress Disorder Among Survivors of Violence and Persecution

**DOI:** 10.7759/cureus.84358

**Published:** 2025-05-18

**Authors:** Mohammad S Ahsan, Ahsan A Sarkar, Md. Al-Amin Khan, Tanbir Ahmed, Mohammed R Islam, Samina Akter, Mrittunjoy K Sarder, Md. Noor -E- Alam, M. Moniruzzaman, Syeda R Jahan, S. M. N Huda, Mahibur R Mubin, Mst. S Sultana, Anamika Sarker

**Affiliations:** 1 Psychiatry, Bangladesh Medical University, Dhaka, BGD; 2 Psychiatry, Pabna Mental Hospital, Pabna, BGD; 3 Child and Adolescent Psychiatry, Bangladesh Medical University, Dhaka, BGD; 4 Urology, Bangladesh Medical University, Dhaka, BGD; 5 Oral and Maxillofacial Surgery, Dhaka Medical College and Hospital, Dhaka, BGD

**Keywords:** bangladesh, depression, persecution, ptsd (post-traumatic stress disorder), revolution, structural violence, survivor, ­trauma

## Abstract

Introduction

This study examined the mental health consequences of Bangladesh’s July Revolution (2024) by assessing the occurrence of depression and post-traumatic stress disorder (PTSD) symptoms among survivors of state violence and persecution while identifying associated risk factors.

Materials and methods

A hospital-based cross-sectional study was conducted from September 2024 to February 2025 among 217 injured survivors (mean age: 26.0 ± 9.7 years; 97.2% male). Participants were assessed via face-to-face interviews using validated tools: Patient Health Questionnaire-9 (PHQ-9) (cutoff: ≥10 for depression) and PTSD Checklist for DSM-5 (PCL-5) (cutoff: ≥33 for PTSD). Univariate logistic regression was performed to analyze sociodemographic and clinical associations.

Results

Depression and PTSD were alarmingly high: 82.5% met the threshold for depression (PHQ-9 mean: 14.7 ± 5.6) and 64.1% for PTSD (PCL-5 mean: 37.6 ± 16.1). Urban survivors had significantly lower odds of depression (OR = 0.47, 95% CI: 0.22-1.00) and PTSD (OR = 0.52, 95% CI: 0.29-0.92) than rural counterparts. Strikingly, 99.3% of PTSD cases had comorbid depression, with strong symptom correlation (r = 0.772, p < 0.001). No other sociodemographic factors (age, sex, education, occupation, and socioeconomic status) or violence type showed significant associations.

Conclusions

The revolution’s survivors exhibited extreme mental health burdens, underscoring an urgent need for trauma-integrated care, especially in rural areas.

## Introduction

The psychological impact of trauma associated with natural disasters tends to diminish over time, whereas trauma resulting from interpersonal violence often persists for years, manifesting as depression, anxiety, somatization, post-traumatic stress disorder (PTSD), and substance abuse [1]. Revolutions, particularly those involving mass violence, can generate large-scale interpersonal trauma.

In political science, a revolution is defined as a transformation in the social and political structure of a state through irregular, extraconstitutional, or violent means, typically driven by mass mobilization and followed by significant social, economic, and cultural changes [2]. This distinguishes revolutions from protests, coups, civil wars, and rebellions.

In 2024, students and job seekers in Bangladesh initiated a peaceful movement against the government’s unfair 30% quota system. This protest (lasting from July 16 to August 5, 2024) escalated into a nationwide revolution, culminating in the fall of the government and ushering in sweeping societal changes. In an attempt to suppress the movement, the government employed extreme repressive measures, including unprecedented deployment of military, paramilitary forces, and police, alongside party-affiliated armed goons.

The crackdown involved live fire, shoot-on-site orders, aerial shooting, mobile tracking, internet shutdowns, curfews, denial of medical care, mass arrests, arbitrary detentions, and enforced disappearances. These actions resulted in an estimated 1,500 deaths, primarily among students and young protesters; over 20,000 injuries; 400 cases of vision loss; and more than 11,000 arrests - the highest toll in Bangladesh since the 1971 Liberation War [3-6]. UNICEF reported an unprecedented rise in psychosocial care demand through its child helpline [6]. The long-term psychological impact of this violent upheaval on survivors remains a critical area for investigation.

The mental health impact of protests, riots, and revolutions is well documented, with studies reporting increased rates of depression, anxiety, PTSD, substance use, and, in some cases, suicidal behavior [7,8]. However, most of these studies were focused on the general population, residents of affected neighborhoods, general patients seeking service from hospitals and clinics, or schoolchildren in impacted areas. Few studies specifically examined the psychological impact on protesters and direct victims. The effects on the general population differ from those on individuals who were wounded or directly victimized during a revolution [1].

Notably, most participants and injured survivors of this revolution were young students from academic institutions. Research indicates that older individuals tend to exhibit greater psychological resilience than younger age groups [9]. Furthermore, despite various advancements, Bangladesh’s general and mental healthcare system faces significant challenges, including insufficient budget allocation, workforce shortages, inadequate training, poor quality of care, lack of comprehensive care, and high out-of-pocket expenses [10,11]. Seeking treatment is often described as a frustrating experience.

Given this context, it is crucial to assess the mental health impact of a revolution on survivors, particularly young individuals navigating a deficient healthcare system. With this background, we aimed to examine the mental health impact of the July Revolution on survivors of violence and persecution. Firstly, we aimed to determine the proportion of survivors exhibiting clinically significant symptoms of depression and PTSD. Secondly, we sought to describe the severity patterns of these conditions. Lastly, we aimed to identify sociodemographic and clinical factors associated with the presence of depression and PTSD symptoms.

## Materials and methods

Study design and sample

**Figure 1:**
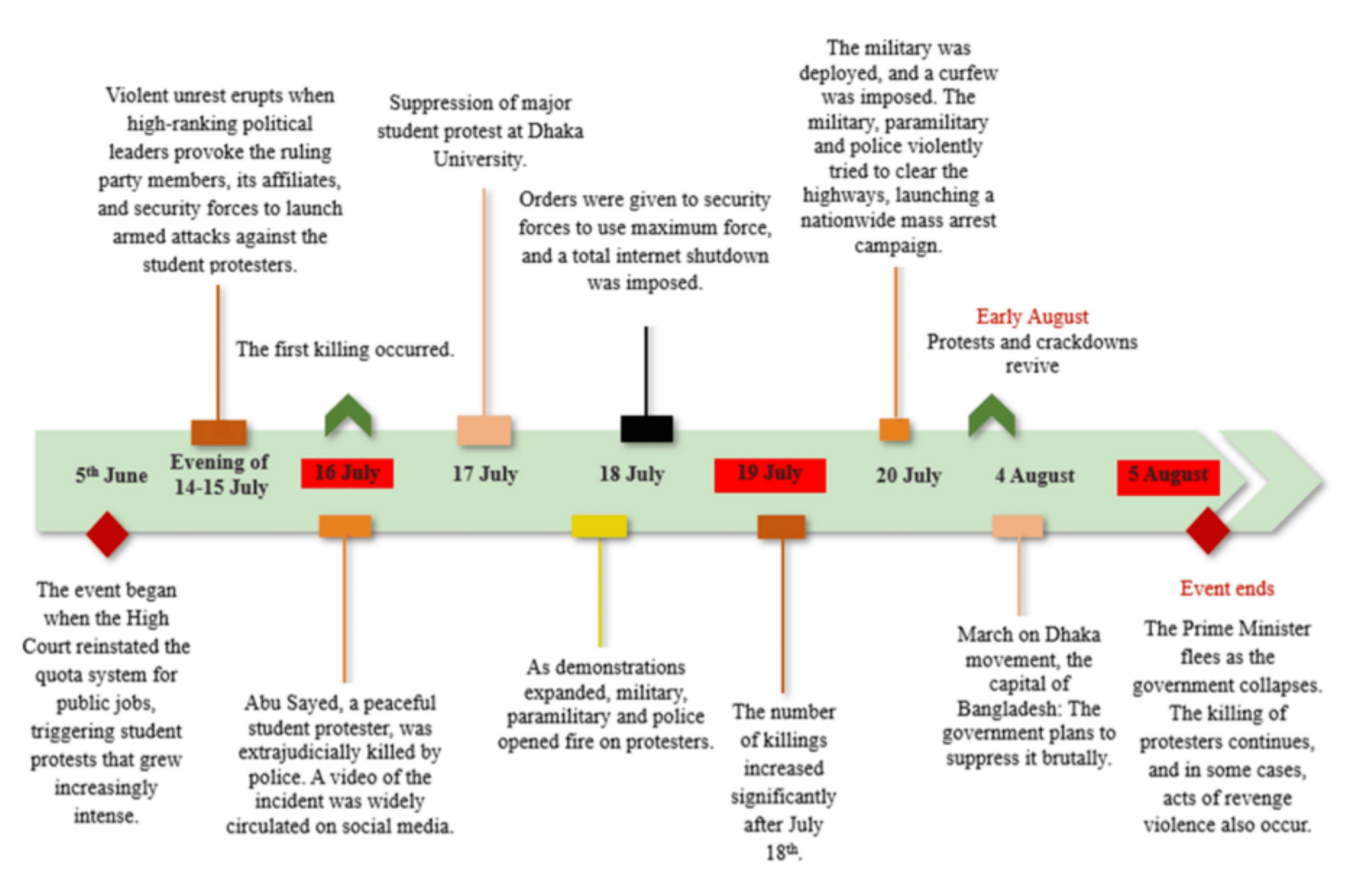
Timeline of major events during the July Revolution in Bangladesh Timeline of specific incidents and the escalation of repression based on the OHCHR Fact-Finding Report [3]. Illustration credit: Tanbir Ahmed

This hospital-based cross-sectional study conveniently enrolled survivors of violence and persecution of the July revolution taking treatment at government hospitals in Dhaka city of Bangladesh within a defined timeframe. Major events of the July Revolution are depicted in Figure 1.

Patient enrollment began in September 2024, one month after the fall of the government, and continued until February 2025. Survivors were recruited from three tertiary hospitals: the National Institute of Ophthalmology & Hospital (NIOH), the National Institute of Traumatology and Orthopaedic Rehabilitation (NITOR), and Bangabandhu Sheikh Mujib Medical University (BSMMU). Each hospital primarily treated specific types of injuries: NIOH predominantly managed cases of ocular trauma, NITOR treated patients with gunshot wounds involving bone injuries and amputations, and BSMMU handled more complex cases requiring advanced medical care.

The sample distribution included 75 participants from NIOH, 65 from NITOR, and 77 from BSMMU. Of the enrolled 217 patients, 214 (98.6%) were receiving medical care, while three (1.4%) were receiving psychological care.

Individuals were eligible for inclusion if they were receiving medical or psychological care at the study hospitals for injuries related to the July Revolution. Exclusion criteria included those unable to communicate due to the severity of their injuries, patients in intensive care units, and individuals who refused to give consent due to concerns about future reprisal.

Measures

Data were collected through face-to-face interviews conducted by psychiatrists using paper-and-pencil methods. For participants who were unable to read, researchers verbally administered the tools by describing each item and recording the responses.

Three tools were used. The first was a semi-structured demographic and clinical questionnaire designed to collect participants’ demographic and clinical information.

The second tool was the Bangla version of the Patient Health Questionnaire-9 (PHQ-9), which was used to screen for depression and assess its severity [12,13]. This nine-item scale uses a 0-3 Likert format, where 0 indicates “not at all” and 3 indicates “nearly every day,” based on symptoms experienced over the past two weeks. A cutoff score of ≥10 has been shown to offer high diagnostic accuracy, with a pooled sensitivity of 74% and specificity of 89% in hospital-based populations for identifying major depressive episodes [14]. The Bangla-validated version demonstrated strong psychometric properties, including good internal consistency (Cronbach’s α = 0.837) and excellent split-half reliability (Guttman split-half coefficient = 0.848). Principal component analysis confirmed a unidimensional structure, with a single factor accounting for 43.9% of the total variance [13]. Depression severity was categorized as follows: 0-4 (none/minimal), 5-9 (mild), 10-14 (moderate), 15-19 (moderately severe), and 20-27 (severe).

The third tool was the PTSD Checklist for DSM-5 (PCL-5), a 20-item self-report measure used to screen for PTSD and provide a provisional diagnosis [15,16]. According to the DSM-5, PTSD involves exposure to actual or threatened death, serious injury, or sexual violence, followed by intrusion symptoms, persistent avoidance, and negative alterations in cognition and mood lasting more than one month. In children under six, PTSD often presents differently, with more frightening dreams, repetitive trauma-themed play or enactment, regressive behaviors, pseudo-hallucinations, and paranoid ideation. Younger children are more likely to display emotional symptoms rather than negative beliefs and are less likely to engage in reckless or self-destructive behavior compared to older individuals.

The PCL-5 items are grouped into four clusters aligned with DSM-5 criteria and are scored on a 0-4 Likert scale, ranging from “not at all” to “extremely,” based on symptoms experienced in the past month. A cutoff score of 31-33 was used for a provisional PTSD diagnosis [17]. The Bangla-validated version of the PCL-5 demonstrated excellent internal consistency (Cronbach’s α = 0.90), strong convergent validity with the PHQ-9 (r = 0.69, p < 0.001), and confirmed the four-factor DSM-5 model through confirmatory factor analysis [16]. PTSD severity was categorized using the scoring criteria provided by Renyer (2016) [18].

Statistical analysis

Data analysis was conducted using IBM SPSS Statistics for Windows, Version 23.0 (Released 2015; IBM Corp., Armonk, NY, USA). Descriptive statistics, including percentages, means, and SDs, were used to summarize the data. Symptom severity was reported as percentages. Depression and PTSD were identified using cutoff scores of ≥10 on the PHQ-9 and ≥33 on the PCL-5. Univariate logistic regression was performed to examine associations between independent variables and depression and PTSD. To ensure adequate sample size within categories, some variables (education, occupation, socioeconomic status, and nature of violence) were collapsed into broader categories before regression analysis.

Ethical considerations

This study adhered to international ethical standards (Declaration of Helsinki). Formal ethics approval was obtained from the Ethics Committee of BSMMU (approval number BSMMU/2024/11983). All data were collected anonymously, and written informed consent was obtained from participants after explaining all relevant details. For children, written informed consent was taken from their guardians and informed assent from them. Participants exhibiting significant distress were referred promptly to available mental health support services to ensure appropriate care and follow-up. All procedures prioritized confidentiality and the psychological well-being of survivors.

## Results

Characteristics of the survivors

A total of 217 injured survivors were enrolled from different study hospitals. The mean (SD) age of participants was 26.0 (9.7) years, with a range of 10 to 67; 12% of the injured were aged 17 or younger. Table 1 presents the demographic and clinical characteristics of the 217 survivors of violence and persecution. The majority were aged 20-29 years (47.9%), most were male (97.2%), and students comprised the largest occupational group (38.7%). Most participants belonged to the lower-middle socioeconomic class (69.6%). The predominant form of violence faced was gunshot wounds (89.4%). A majority (81.1%) received timely treatment, while 10.1% experienced delays exceeding 24 hours.

**Table 1:** Characteristics of the survivors of violence and persecution in the July Revolution, Bangladesh (N = 217) Other occupations included various daily wage earners. Other forms of violence included chemical attacks and property destruction.

Characteristics	Category	n (%)
Age (year)	10-19	54 (24.9)
	20-29	104 (47.9)
	≥30	59 (27.2)
Sex	Male	211 (97.2)
	Female	6 (2.8)
Place of residence	Urban	123 (56.7)
	Rural	94 (43.3)
Educational level	No formal education	8 (3.7)
	Primary	56 (25.8)
	Secondary	82 (37.8)
	Higher secondary	43 (19.8)
	Graduation	28 (12.9)
Occupation	Student	84 (38.7)
	Service	25 (11.5)
	Business	28 (12.9)
	Housewife	1 (.05)
	Unemployed	8 (3.7)
	Others	80 (36.8)
Socioeconomic status	Low	30 (13.8)
	Lower-middle	151 (69.6)
	Upper-middle	9 (4.1)
	Upper	27 (12.4)
Nature of violence	Gunshot wound	194 (89.4)
	Beating	14 (6.5)
	Arrest	1 (0.5)
	Forced search	2 (0.9)
	Others	6 (2.8)
Treatment delay	No delay	176 (81.1)
	Within 24 hours	19 (8.8)
	≥24 hours	22 (10.1)

Burden of depression and PTSD

Based on the established cutoff points, 82.5% of survivors met the criteria for depression. The mean (SD) PHQ-9 score was 14.7 (5.6), with a range of 0 to 26. Similarly, 64.1% of survivors met the criteria for a provisional PTSD diagnosis. The mean (SD) PCL-5 score was 37.6 (16.1), with a range of 1 to 75. Table 2 presents the distribution of participants across different severity levels on the PHQ-9 and PCL-5 scales. The majority of participants fell into the moderate to severe depression category and the moderate to severe PTSD category.

**Table 2:** Distribution of survivors by depression (PHQ-9) and PTSD (PCL-5) severity levels (N = 217) PCL-5, PTSD Checklist for DSM-5; PHQ-9, Patient Health Questionnaire-9; PTSD, post-traumatic stress disorder

Depression	n (%)	PTSD	n (%)
None/minimal	15 (6.9)	Normal	46 (21.2)
Mild	23 (10.6)	Mild	32 (14.7)
Moderate	57 (26.3)	Moderate	86 (39.6)
Moderately severe	79 (36.4)	Severe	44 (20.3)
Severe	43 (19.8)	Extremely severe	9 (4.1)
Depression (≥10 on the PHQ-9)	179 (82.5)	PTSD (≥33 on the PCL-5)	139 (64.1)

Associations of depression and PTSD

Table 3 presents the results of univariate logistic regression analysis examining the association between sociodemographic and clinical factors with depression and PTSD among survivors. Residence was significantly associated with both depression and PTSD, with urban survivors having lower odds of depression (OR = 0.47; 95% CI: 0.22-1.00, p = 0.049) and PTSD (OR = 0.52; 95% CI: 0.29-0.92, p = 0.027) compared to rural survivors.

**Table 3:** Association of sociodemographic and clinical factors with depression and PTSD among survivors of violence and persecution determined by univariate logistic regression ^*^ p < 0.05 Non-students included service holders, business owners, housewives, unemployed individuals, and various daily wage earners. Other forms of violence included beatings, arrests, forced searches, chemical attacks, and the destruction of property. PTSD, post-traumatic stress disorder

Characteristics	Category	Depression			PTSD		
		OR (95% CI)	Wald χ²	p-value	OR (95% CI)	Wald χ²	p-value
Age (year)	10-19	0.42 (0.15-1.16)	2.78	0.095	0.63 (0.29-1.38)	1.28	0.257
	20-29	0.64 (0.25-1.64)	0.85	0.357	0.76 (0.38-1.51)	0.6	0.436
	≥30	1	-	-	1	-	-
Sex	Male	2.43 (0.42-13.77)	1	0.316	1.81 (0.35-9.20)	0.51	0.473
	Female	1	-	-	1	-	-
Place of residence	Urban	0.47 (0.22-1.00)	3.76	0.049*	0.52 (0.29-0.92)	4.88	0.027*
	Rural	1	-	-	1	-	-
Educational level	No formal education and primary	1.47 (0.47-4.54)	0.45	0.501	1.65 (0.67-4.08)	1.19	0.275
	Secondary	1.12 (0.39-3.23)	0.04	0.827	1.58 (0.66-3.78)	1.07	0.3
	Higher secondary	1.68 (0.48-5.86)	0.66	0.414	1.79 (0.67-4.77)	1.37	0.241
	Graduation	1	-	-	1	-	-
Occupation	Student	0.64 (0.32-1.31)	1.44	0.23	0.85 (0.48-1.51)	0.27	0.6
	Non-students	1	-	-	1	-	-
Socioeconomic status	Low	0.64 (0.17-2.37)	0.43	0.509	0.84 (0.31-2.30)	0.1	0.746
	Lower-middle	0.74 (0.26-2.07)	0.32	0.569	1.04 (0.49-2.23)	0.01	0.909
	Upper-middle and upper	1	-	-	1	-	-
Nature of violence	Gunshot wound	1.78 (0.65-4.88)	1.28	0.258	1.42 (0.59-3.42)	0.62	0.428
	Others	1	-	-	1	-	-
Treatment delay	No delay	1.90 (0.68-5.26)	1.52	0.201	3.09 (1.24-7.66)	5.95	0.015*
	Within 24 hours	2.00 (0.42-9.41)	0.76	0.381	1.60 (0.46-5.53)	0.56	0.454
	≥24 hours	1	-	-	1	-	-

Those who received treatment without any delay had 3.09 times higher odds of PTSD compared to those experiencing delayed treatment (p = 0.015). No significant associations were found for age, sex, education level, occupation, socioeconomic status, or nature of violence with depression or PTSD.

Among the 139 survivors with a provisional PTSD diagnosis, 138 (99.3%) had comorbid depression, and similarly, among the 179 survivors with depression, 138 (77.1%) also met the criteria for PTSD (OR = 124.5; 95% CI: 16.5-935.6, p < 0.001). The PHQ-9 and PCL-5 scores showed a strong positive correlation (r = 0.772, p < 0.001).

## Discussion

Survivors

This study examined the burden and correlates of depression and PTSD among survivors of the July Revolution in Bangladesh who directly experienced violence and persecution. The demographic profile of the survivors indicates that they were predominantly young, male, well-educated individuals from lower-middle-income backgrounds. This demographic pattern provides insight into the composition of those who participated in the revolution. Historically, revolutions have been driven primarily by youth, as young people are more likely to engage in social and political movements due to their idealistic perspectives, fewer familial responsibilities, feelings of marginalization, and unemployment. Additionally, revolutions are more likely to succeed when young individuals actively participate [19].

The predominance of male participants aligns with the sociopolitical context of the Indian subcontinent, where patriarchal societal structures, cultural norms, and gender inequality contribute to lower female participation in political movements [20]. Notably, during the initial peaceful phase of the movement, a substantial number of women were involved. However, as the situation escalated into violence, men became the dominant presence on the streets.

The significant representation of students and well-educated individuals from lower-middle-income backgrounds reflects the broader economic challenges facing Bangladesh. While academic enrollment rates have increased and the population remains predominantly young, limited access to capital and financial resources restricts career opportunities. For many, securing formal employment is the only viable means of socioeconomic mobility. The quota system in government employment, perceived as a barrier to merit-based advancement, has been a particular source of frustration for educated youth from lower-middle-class backgrounds.

Interestingly, a notable number of individuals from upper-class backgrounds also participated in the revolutionary movement. This may be understood through the psychological concept of deindividuation, wherein individuals, acting within a collective, prioritize group identity over personal identity. In this case, the identity of being a student or protester may have overridden socioeconomic distinctions [21]. The equal representation of urban and rural participants suggests that the movement had widespread appeal across different population groups.

Regarding the nature of violence experienced, gunshot wounds were the most commonly reported injuries, aligning with media reports indicating that 78% of fatalities during the revolution were due to gunshot wounds [22]. Importantly, despite the high levels of psychological distress among survivors, the overwhelming majority (98.6%) sought medical care for their physical injuries, with only a small fraction receiving psychological support. This suggests a lack of mental health awareness, both among survivors and healthcare professionals, regarding the profound mental health consequences of severe physical trauma.

Main findings

The findings of this study indicate a high burden of mental health disorders among survivors, with 82.5% meeting the criteria for depression and 64.1% receiving a provisional diagnosis of PTSD. Comparatively, a systematic review examining the impact of communal riots on the general population and affected communities reported a prevalence of depression at 49% and PTSD ranging from 4% to 41% [8]. Another systematic review of 52 studies found a 7% increase in depression prevalence following exposure to violence, with PTSD prevalence ranging between 4% and 41% [7]. Among Rwandan genocide survivors, the prevalence of PTSD and depression was reported to be 46% for both conditions [23]. The significantly high rates of depression and PTSD observed in this study may be attributed to several factors, including exposure to life-threatening injuries, direct involvement in life-threatening situations, the occurrence of permanent bodily injuries, the need for prolonged medical care, witnessing the trauma and suffering of fellow protesters, and a lack of prior experience in coping with extreme repression, compounded by the absence of psychiatric care.

Additionally, the elevated symptom levels may reflect a referral bias, as individuals with more severe symptoms are more likely to seek care at tertiary hospitals. The study also identified a significant association between place of residence and mental health outcomes, with rural survivors exhibiting higher odds of both depression and PTSD compared to their urban counterparts. This disparity may be explained by differences in access to mental health services, social support networks, and economic stability, which are often more limited in rural areas [24]. Urban survivors may have benefited from better healthcare infrastructure and community resources, which could mitigate psychological distress, whereas rural populations may have faced prolonged exposure to trauma with limited access to psychological support and intervention.

An unexpected finding was the increased likelihood of PTSD among survivors who received immediate treatment compared to those who experienced delays in care. While counterintuitive, this pattern may suggest that individuals who sought early treatment had been exposed to more severe trauma, prompting quicker help-seeking behavior. This interpretation aligns with evidence from a meta-analysis of 14 risk factors for PTSD development, which identified severity of traumatic experience as one of the strongest predictors of PTSD, along with lack of social support and continuing life stress [25].

The high comorbidity between depression and PTSD observed in this study is consistent with existing literature that highlights the frequent co-occurrence of trauma-related disorders and mood disorders. Many symptoms of PTSD and depression overlap, such as low mood, loss of interest, irritability, guilt, inattention, sleep disturbances, and thoughts of death [26]. In the absence of a formal clinical diagnosis, screening tools often yield overlapping diagnoses due to the shared symptomatology between these conditions. The extremely high OR emphasizes the interconnected nature of these conditions, suggesting that trauma survivors are at a significantly increased risk of developing both disorders simultaneously. This finding aligns with previous studies, which have reported high comorbidity rates among PTSD patients, ranging from 60% to 98.8% [27].

Interestingly, sociodemographic factors such as age, sex, education, occupation, and socioeconomic status did not significantly predict depression or PTSD in this sample. This contrasts with some prior studies where these variables were influential [7], potentially suggesting that the widespread exposure to extreme violence during the July Revolution may have overshadowed individual differences. This interpretation is consistent with findings from a meta-analysis, which concluded that factors occurring during or after the traumatic event, such as the severity of trauma, level of social support, and ongoing life stress, are more predictive of PTSD development than pre-trauma factors like younger age, female sex, low education, low intelligence, lower socioeconomic status, previous psychiatric history, childhood abuse, previous trauma, or family history of psychiatric illness [25]. Furthermore, PTSD prevalence following intentional trauma tends to increase over time, in contrast to non-intentional trauma (e.g., natural disasters), where symptom prevalence typically declines [28].

Additionally, the nature of the violence (e.g., gunshot wound vs. others) did not differentiate risk, which may be attributed to the small sample size of individuals experiencing other injuries. It is possible that the small sample size of individuals who experienced other injuries could limit the ability to detect significant differences in PTSD and depression risk related to the nature of the violence.

Strengths and limitations

This study provides valuable insights into the mental health consequences of the July Revolution in Bangladesh by focusing on a rarely studied population - injured survivors of a revolutionary movement. The use of validated screening tools (PHQ-9 and PCL-5) enhances the reliability of depression and PTSD assessments, while the examination of sociodemographic factors offers a preliminary understanding of risk in this context. However, several limitations must be acknowledged. The cross-sectional design restricts the ability to infer causal relationships between exposure to state violence and the development of depression or PTSD. The use of convenience sampling within hospital settings may introduce selection bias, limiting the generalizability of findings to the broader population of survivors. Logistical challenges, such as the absence of centralized medical records and poor coordination among healthcare facilities, constrained the sample size and may have influenced participant recruitment. Additionally, the study did not assess critical clinical variables such as the severity and nature of physical injuries, the specific bodily areas affected, levels of disability, or quality of life - factors that could influence mental health outcomes. While validated screening instruments (PHQ-9 and PCL-5) were used to assess depression and PTSD symptoms, these tools provide provisional, not diagnostic, measures and should be complemented by structured clinical interviews in future research.

Implications of the findings

This study highlights the urgent need for integrated trauma care addressing the strong depression-PTSD comorbidity, particularly for underserved rural survivors. Policy-wise, establishing national trauma rehabilitation programs, centralized survivor registries, and mental health awareness campaigns is critical. Future research should employ longitudinal designs, incorporate injury severity and disability data, and explore resilience factors to better inform culturally adapted interventions.

## Conclusions

This study reveals the profound psychological toll of political violence, demonstrating high comorbidity of depression and PTSD among survivors of Bangladesh’s July Revolution, regardless of most sociodemographic factors. Rural residence was significantly associated with poorer mental health outcomes, highlighting disparities in access to mental health support. Variables such as age, education, and economic status were not significant predictors, likely due to the overwhelming and shared traumatic exposure. These findings underscore the urgent need for trauma-informed mental health services and long-term support systems for survivors. Without targeted interventions, the enduring mental health consequences may impede both individual recovery and broader societal healing.
